# Feasibility of ROBUST: An Integrated Sensorimotor Upper Limb Therapy for People in the Chronic Phase After Stroke

**DOI:** 10.3390/brainsci16080844

**Published:** 2026-08-08

**Authors:** Charlotte Heremans, Inês Alves Martins, Siqi Yang, Jolien Gooijers, Jean-Jacques Orban de Xivry, Geert Verheyden

**Affiliations:** 1 Department of Rehabilitation Sciences, KU Leuven, 3001 Leuven, Belgium; 2 Leuven Brain Institute, KU Leuven, 3000 Leuven, Belgium; 3 Department of Movement Sciences, KU Leuven, 3001 Leuven, Belgium

**Keywords:** chronic stroke, upper limb, sensorimotor therapy, robotic, home program, sensory processing, feasibility

## Abstract

**Highlights:**

**What are the main findings?**

- Integrated sensorimotor upper limb therapy (combining robotic, therapist-led, and home-based therapy) is feasible.
- The approach may improve clinical, kinematic and patient-reported outcomes in chronic stroke.

**What are the implications of the main findings?**

- A pilot clinical trial is warranted to assess effectiveness.
- The approach may enable more intensive, integrated rehabilitation.

**Abstract:**

**Background/Objectives**: Upper limb rehabilitation in chronic stroke is often suboptimal, and somatosensory integration is frequently neglected. The aim of this study was to investigate the feasibility and potential effects of an integrated sensorimotor therapy. **Methods**: Individuals with chronic stroke and persistent sensorimotor and functional upper limb deficits were included. For four weeks, participants received standard care (control), followed by 48 h of integrated sensorimotor upper limb therapy (ROBUST) over four weeks in addition to their standard care (intervention). ROBUST combined robotic, therapist-led, and home exercises. Feasibility was assessed through adherence, motivation, experiences, and adverse events. Potential effects of the intervention were evaluated via a multimodal assessment approach at baseline (T1), post-control (T2), and post-intervention (T3), with the Action Research Arm Test (ARAT) as the primary outcome. **Results**: Ten participants were included in this study, of whom nine completed the protocol. Therapy session adherence was 100%, and home program adherence 67%. Motivation remained stable over the intervention period, participants were positive, and no adverse events occurred. Six participants (67%) improved more than the minimal clinically important difference for ARAT, with further relevant improvements in clinical, kinematic, and patient-reported outcomes observed. **Conclusions**: The novel integrated sensorimotor upper limb therapy (ROBUST) is feasible and shows promising potential for people after stroke in the chronic stage. A randomised controlled trial is warranted. **Trial registration**: (identifier: NCT06870682).

## 1. Introduction

Stroke is the leading cause of adult disability in Europe [1], with approximately two-thirds of patients with stroke experiencing significant upper limb impairments [2]. Therefore, effective upper limb rehabilitation remains essential, even in the chronic phase (>6 months post-stroke). Although the first three months post-stroke are considered a “critical window” for recovery due to heightened neuroplasticity [3,4], research has shown that responsiveness to treatment extends beyond this period, with meaningful improvements possible after that “critical window” [5,6,7,8,9]. In the chronic phase, neuroplasticity is primarily driven by therapeutic input, as spontaneous recovery has generally plateaued [10,11], highlighting the importance of targeted and effective rehabilitation strategies months after the stroke.

Recent work by Purton et al. [12] demonstrated that individuals with chronic stroke prioritise upper limb rehabilitation over lower limb rehabilitation during the chronic phase because of the challenges they address once coming home. However, current rehabilitation practices often emphasise lower limb therapy during the chronic phase, thus failing to meet this need [12]. The current insufficient or ineffective upper limb therapy provision in the chronic phase is also suggested by the 5-year follow-up results reported by Meyer et al. [13], showing that arm function and overall functionality at five years post-stroke are comparable to levels at two months post-stroke.

Somatosensory function is required during many daily activities (e.g., washing hair, buttoning a shirt) [14,15]. Yet, somatosensory rehabilitation is frequently overlooked in chronic stroke care [16,17]. Somatosensory deficits can significantly impact dexterity, motor function, coordination, and thereby participation, functionality, and quality of life [18,19,20,21,22]. Despite this [16,17,18,19,20,21,22,23], evidence supporting somatosensory-focused therapy remains limited, and clinicians increasingly advocate for a shift in treatment approaches [24]. Furthermore, Van Gils et al. [25] found that sensory processing is a predictor for moderate to good bimanual outcome in people with stroke. Sensory processing is a sensory modality that requires higher-order cortical functions enabling discrimination of objects, structures, positions and movements. Thus, there is a need for somatosensory interventions, especially with a focus on sensory processing, within rehabilitation protocols [22,26].

Carey et al. [27] investigated the effectiveness of a sensory processing program based on the SENSe principles (Study of the Effectiveness of Neurorehabilitation on Sensation) compared to non-specific repeated exposure to sensory stimuli via passive movements and grasping of common objects. The SENSe principles emphasise intensive, repetitive, and tailored practice; graded exercises of increasing difficulty, attentive exploration, and calibration to better transfer somatosensory improvement to daily activities [28]; and are recommended by the Australian National Stroke Guidelines [28]. The sensory processing program consisted of three tasks: texture discrimination, limb position sense (ability to perceive the position and movement of the upper limb without using vision), and tactile object recognition. They found that the group receiving the sensory processing program showed a significantly greater improvement in sensory processing ability compared to the group receiving only the repeated exposure to sensory stimuli, and the improvement was also maintained after a follow-up of six weeks and six months.

However, a systematic review highlighted that isolated somatosensory training is ineffective in improving upper limb motor impairment and activity [29], suggesting that integrated sensorimotor therapy may be more beneficial. Therefore, as a next step, De Bruyn et al. [20] combined the sensory processing program of Carey et al. [27] with a newly developed sensorimotor training program, whereby task-specific upper limb exercises were carried out with sensory integration, such as reaching toward different structures. These sensorimotor therapy tasks were also based on the SENSe principles [30]. De Bruyn et al. [20] found no significant difference between the group receiving the sensorimotor therapy and the group receiving motor therapy only for the Action Research Arm Test (ARAT) or sensory measures. For the Fugl–Meyer Assessment for Upper Extremities (FMA-UE), a significantly greater improvement was found for the motor group. A possible reason why the sensorimotor therapy of De Bruyn et al. [20] showed no additional benefit over motor training might be that the sensorimotor exercises were delivered only in a conventional manner, without the inclusion of robotics, thereby limiting their intensity. In fact, a recent systematic review showed that robot-assisted therapy is more effective for upper limb outcomes than therapy that does not use robotic devices. More specifically, the review found that end-effector robotics provided the greatest benefit [31]. Saenen et al. [32] developed robotic SENSe-based sensorimotor therapy tasks. In this pilot study, including ten participants with chronic stroke, ten one-hour sessions over four weeks resulted in significant motor (effect size = 0.79) and sensorimotor (effect size = 0.72) improvements, although these changes did not reach the MCID [32]. The absence of functional training (i.e., training with daily life objects based on personalised goals of the participants) in the training protocol may have contributed to the limited clinical gains.

Home training can offer the inclusion of functional training. A systematic review and meta-analysis found that home rehabilitation can improve functional outcomes in stroke survivors [33]. Simpson et al. [34] tested the Home-Graded Repetitive Arm Supplementary Program (H-GRASP) in eight participants with chronic stroke and suggested improvements in perceived upper limb activity. This program was adapted and investigated in a larger sample by Essers et al. [35] (AH-GRASP), who found that 48 h of AH-GRASP resulted in positive patient experiences and significant improvements in upper limb capacity (effect size = 0.26), self-efficacy (effect size = 0.52), and contribution of the affected upper limb to overall activity and performance (effect size = 0.64). Furthermore, using the H-GRASP program is strongly recommended in the Canadian Stroke Best Practice Recommendations [36]. Despite these promising results, adherence remains a challenge. In the study of Essers et al. [35], only 41% of participants met the prescribed exercise target (12 h/week, 4 weeks).

Given the complementary strengths and limitations of therapist-led, robotic, and home-based sensorimotor interventions [20,32,35], the current study aimed to investigate the feasibility and possible effects of a combined therapy approach called ROBUST (integrated ROBotic Upper limb sensorimotor rehabilitation after STroke), in individuals with chronic stroke. To assess feasibility, we gathered information regarding adverse events, adherence and participants’ experiences. To evaluate potential effectiveness, we used a multi-level assessment protocol, including clinical assessments, kinematic measurements, and a patient-reported outcome. We hypothesised that ROBUST is feasible and potentially effective, based on the results of the individual therapy interventions [20,32,35].

## 2. Materials and Methods

### 2.1. Study Design and Procedure

**Figure 1:**
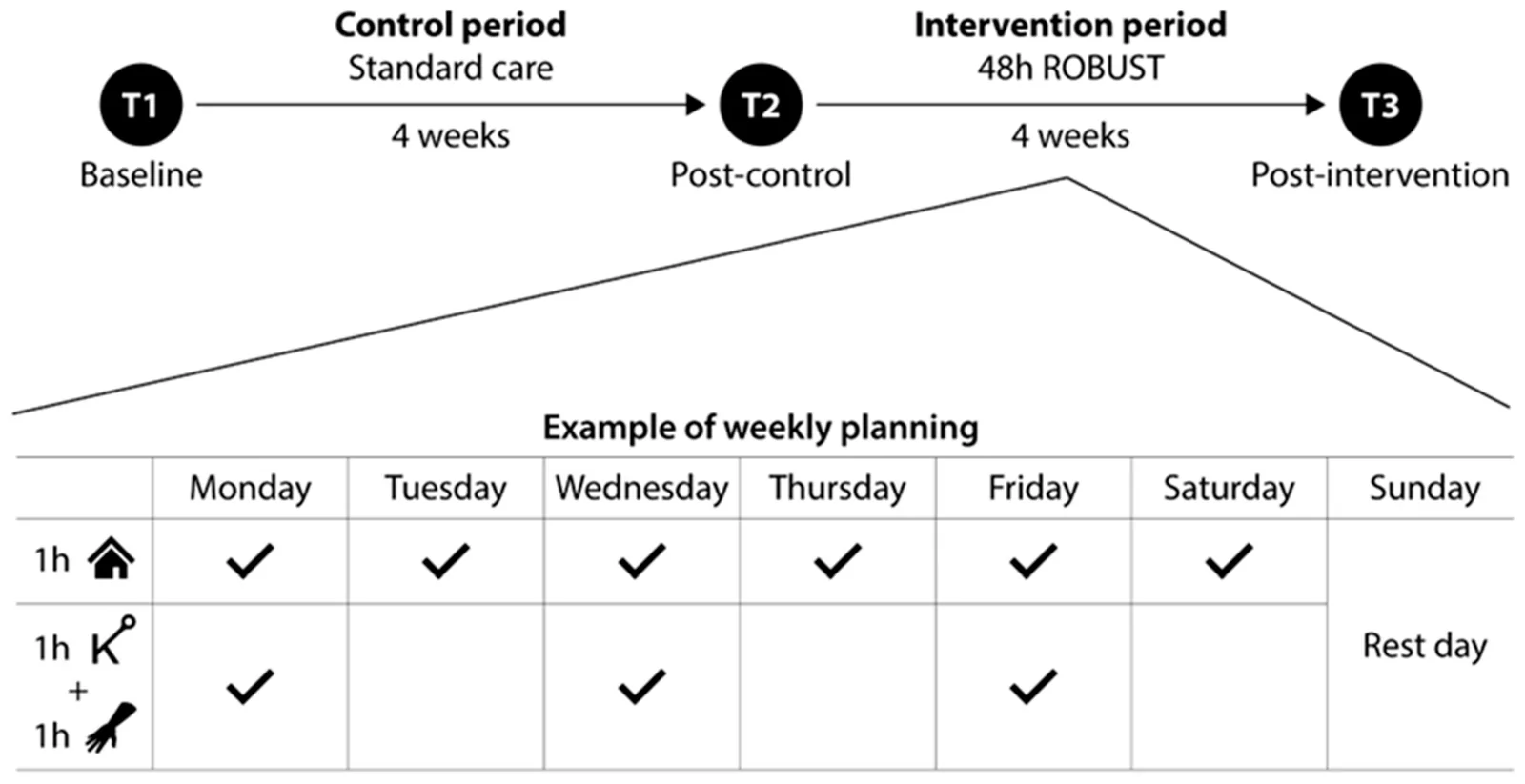
Study design. During the intervention period, participants were required to complete a one-hour home program (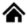) for six days per week, performed independently at home. In addition, three times per week, the participant was required to attend a 2 h therapy session at BrainsHub (Leuven, Belgium), consisting of one hour of robotic therapy (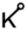) and one hour of therapist-led therapy (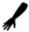). The checkmarks (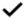) indicate on which days the component of the therapy occurred, for a weekly planning example.

This pilot intervention study consisted of a single-group, pre–post design. During the first four weeks of the study, participants followed their standard care only, which averages 2 h per week of physiotherapy in Belgium. After this control period, participants underwent an additional 48 h combined sensorimotor intervention program, referred to as ROBUST, over four weeks. Standardised assessments were completed at baseline (T1), after the control period (T2), and after the intervention period (T3). An overview of the study design can be found in Figure 1. The assessments, as well as the robotic and therapist-led therapy sessions, took place at BrainsHub (KU Leuven). The home-based therapy sessions were completed independently and unsupervised at the participant’s home. Ethical approval was obtained from the Ethics Committee of UZ/KU Leuven (protocol No. S69003), and the study was pre-registered on Clinicaltrials.gov, accessed on 6 May 2026 (NCT06870682).

### 2.2. Participant Selection

Individuals were eligible if they met all of the following criteria: (1) first-ever supratentorial stroke; (2) age ≥ 18 years; (3) >6 months post-stroke; (4) mild to moderate upper limb motor impairment, defined by a FMA-UE [37] score ≥ 23/66 (subjects with a score < 23/66 were excluded due to inability to handle the robotic device); (5) residual sensory upper limb impairment, defined as Tactile Discrimination Test (TDT) [38] score < 24/25; (6) impaired functionality, defined as Sensorimotor Action Research Arm Test (sARAT) [39] score < 52/57; and (7) written informed consent provided. Exclusion criteria included (1) musculoskeletal disorders in the most affected upper limb; (2) other neurological disorders; (3) severe communication or cognitive deficits interfering with protocol compliance; (4) severe spasticity preventing the use of the robotic device; (5) any other condition compromising safety or participation (e.g., uncontrolled epilepsy); and (6) participation in another clinical investigation.

Interested participants were pre-screened via phone or email to minimise unnecessary travel and effort for those clearly ineligible, based on simple yes/no questions. Individuals who passed this initial screening were invited for an in-person visit, during which informed consent was obtained, and the FMA-UE, the TDT, and the sARAT were administered to confirm eligibility.

### 2.3. Study Periods

#### 2.3.1. Control Period: Standard Care

During the first four weeks, participants received standard care only. The content and dosage of this care varied between individuals, as it reflected the therapy they were already receiving as part of their routine clinical management and was provided by their own healthcare providers. In Belgium, this standard care typically amounts to approximately two hours per week.

#### 2.3.2. Intervention Period: Standard Care + ROBUST

**Figure 2:**
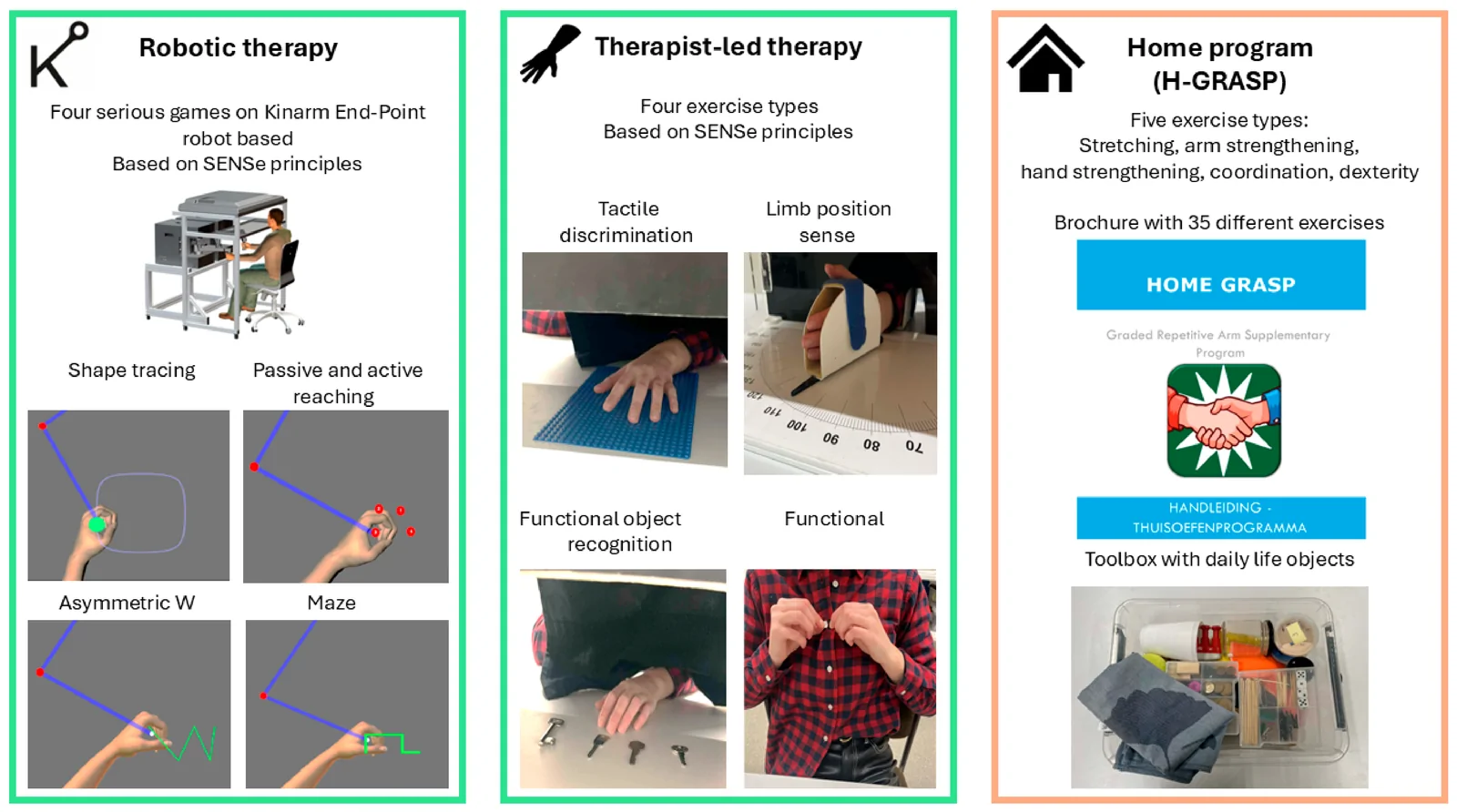
Overview of the different therapy types within ROBUST with examples of the exercises. The green boxes are the therapies provided at BrainsHub (KU Leuven, Belgium). In the “Robotic therapy” box, the blue lines and the red dot connecting them represent the robotic arm’s position, which is not visible to the participants; the remaining elements illustrate the participant’s hand position or trajectory. The orange box is the home program, conducted independently by the participant in their home environment.

During the intervention period, participants received a 48 h ROBUST intervention additional to their standard care over four weeks. The ROBUST intervention consisted of three therapy elements: robotic, therapist-led, and home-based therapy. ROBUST was considered a sensorimotor intervention because several therapy components required the integration of sensory processing and motor execution during task performance. For both the robotic and therapist-led therapy sessions, participants were required to attend three 2 h sessions per week at BrainsHub (Leuven, Belgium). During these 2 h sessions, robotic therapy was provided during the first hour, followed by therapist-led therapy during the second hour. Both sessions were delivered by a researcher with a master’s degree in physiotherapy (CH). In addition to these sessions, participants were required to follow a home program (H-GRASP) for six hours per week. An example of a weekly schedule for a participant is shown in Figure 1, and an overview of the ROBUST intervention can be found in Figure 2. Detailed information can be found below.

##### Robotic Therapy

The robotic therapy was based on Saenen et al. [32] and was provided using the Kinarm End-Point robot (BKIN Technologies Ltd., Kingston, ON, Canada), which is a 2D planar robot allowing for shoulder and elbow movement in the horizontal plane, without anti-gravity support, through active grasping of the end-point handles. This robotic device also includes a virtual reality screen placed above the workspace to allow for visual feedback control. A black cloth was used to prohibit direct vision of the arms. Each session began with a 7 min warm-up, consisting of passive- or active-assisted mobilisations of the upper limb joints, without use of the robotic device. After the warm-up, four robot-assisted serious games focusing on proprioceptive sensory processing and based on the SENSe training principles [30] were practised for 10 min each. During each task, progression was based on the task score and the BORG Rating of Perceived Exertion Scale (Borg RPE) [40], which was completed every five minutes of practice.

For the Shape Tracing Task, participants were asked to keep their most affected hand inside a target that was moving along a path displayed on the screen. Visual feedback of hand position was given intermittently. A colour change of the moving target indicated whether the hand was within (green) or outside (red) the target. The speed of the target was 4 cm/s and difficulty was increased by increasing the complexity of the shape, increasing the interval between periods where the visual feedback was visible or reducing the size of the target, creating a total of 15 levels. After each trial, a score was shown, indicating the percentage of the time that the hand position was within the target. Progression was made when participants achieved a score of 80% or higher for 8 out of 10 trials. This task was developed as the proprioceptive tracing task by Keeling et al. [41] and used by Saenen et al. [32].

The Passive and Active Reaching Task was developed by Saenen et al. [32]. During the first part, the Kinarm robot passively moved the participant’s most affected arm to a target location, beginning from the starting point that was positioned 20 cm in front of the shoulder. The passive movement always had a duration of 2.5 s. After the movement, various targets (red dot with a white number on it) appeared on the virtual screen, one of which corresponded to the correct location of the most affected hand. The participant was then asked to orally indicate the number of the target that was located at the position of the hand in the absence of any visual feedback about hand position. During the second part, the participant was asked to actively return to the starting point. Visual feedback regarding the starting point was only provided when the hand position was within a 5 cm radius of the starting point. When the starting target was not reached within 5 s, assistive forces of maximum 20 Nm were applied. Difficulty was increased by first increasing the number of targets, and then by decreasing the distance between them (distances included 20 cm, 15 cm, 10 cm, 5 cm, and 2.5 cm), creating a total of 19 levels. Progression to the next difficulty was made when at least 8 out of 10 trials were correctly answered (i.e., the correct target number was indicated).

The Asymmetric W Task was an angle discrimination task developed by Saenen et al. [32]. During this task, participants were asked to actively or passively follow the path of an asymmetric W with their most affected arm, without visual feedback. We considered the two angles at the bottom of the W shape (Figure 2). One of these angles was larger than the other one, and the participant had to indicate which angle was the largest. During the passive condition, the Kinarm robot passively moved the participant’s most affected arm along the path of the W shape. In the active condition, the shape was delimited with virtual walls that constrained the hand along the path. The active condition was preferred, but a switch to the passive condition was made whenever a Borg RPE score of ≥15 was indicated by the participant. Task difficulty was increased by reducing the angle difference (from 40° to 10° with decrements of 5°), creating a total of 7 levels. Progression was made when at least 8 out of 10 trials were correctly answered.

The Maze Task was also developed by Saenen et al. [32]. During the first part (the learning phase), participants had to actively or passively follow the path of one of two mazes with their most affected arm, without visual feedback. Thereafter, they were asked to describe and trace the shape of the maze using the handle of the Kinarm robot, after which this was discussed based on visual and verbal feedback. The passive and active conditions were identical to those described for the Asymmetric W Task. Once two mazes were learned, one of the two mazes was readministered, and the participant was asked to indicate which one of the two it was. Task difficulty was increased by increasing the similarity between the two mazes, creating a total of 3 levels. Progression was made when at least 8 out of 10 trials were correctly answered.

A detailed description of each task can be found in Saenen et al. [32]. To conclude the first hour of each session, active neck and scapula mobilisations were performed for three minutes without the use of the robotic device, to avoid muscle stiffness because of sitting in the same position during the robotic tasks.

##### Therapist-Led Therapy

During the second hour of each therapy visit, participants received one-to-one, non-robotic therapy from a physiotherapist. The order of the robotic and therapist-led tasks was fixed, whereby we first trained the sensorimotor function, and thereafter translated these skills to activities involving daily-life objects. This therapist-led therapy consisted of four 15 min segments. During the first three segments, the sensory discrimination tasks described by De Bruyn et al. [20], which are based on the SENSe approach, were trained: texture discrimination, limb position sense, and tactile object recognition [27]. There was a mixture of sensory and sensorimotor exercises. During the last 15 min, functional exercises tailored to the participant’s personalised goals were trained. Before the start of the first therapy session, each participant was asked to identify one to three personal goals. A full list of the personalised goals can be found in Appendix A.

##### Home Program

The home program (H-GRASP) was a task-oriented training involving graded, self-administered exercises, based on the studies of Simpson et al. [34] and Essers et al. [35]. The exercises focused on stretching, strengthening, weight bearing, and fine and gross motor skills. During the first therapy session, all exercises were explained to the participant. Each participant received a booklet containing written instructions with pictures for each exercise, as well as a kit with the necessary materials to complete the exercises.

For most exercises, recommended progressions in repetitions were provided (e.g., three sets of 5, 8, or 10 repetitions). For some exercises, progression could be achieved by modifying the materials used (e.g., larger or smaller blocks). Furthermore, participants were asked to keep a daily log to track the number of repetitions and sets for each exercise, the number of days and total time spent per day completing the exercises, and the perceived difficulty of the exercises (based on a numeric rating scale ranging from 0 to 10).

A number of behavioural strategies, based on the key themes influencing adherence to home-based upper limb practice after stroke identified by Neibling et al. [42], were incorporated. These included identifying barriers to adherence, problem-solving, and setting weekly goals. The strategies were implemented through weekly discussions during one of the therapy visits.

Because ROBUST combines multiple interacting intervention components, the study was not designed to determine the individual contribution of each component. However, a conceptual overview of the hypothesised relationships between intervention components, targeted mechanisms, and outcome measures is presented in Table 1.

### 2.4. Outcomes

#### 2.4.1. Participants’ Characteristics

Demographic and stroke characteristics were recorded at baseline (T1). These included age, gender, time post-stroke, lateralisation of symptoms, and type of stroke.

#### 2.4.2. Feasibility

The feasibility of this novel integrated therapy approach was evaluated using various outcomes, including recruitment rate (percentage of individuals approached who were eligible and agreed to participate), retention rate (percentage of participants who completed the 4-week intervention), adherence to the intervention protocol (percentage of participants achieving the total amount of 48 h additional therapy), safety (occurrence of related adverse events), patient experiences, and reasons for withdrawal.

Based on the previous studies including the robotic, therapist-led and home training [20,32,35], we anticipated a recruitment rate of 31%, a retention rate of 90%, an adherence rate for the therapy sessions of 95%, an adherence rate for the home training of 41%, and no related adverse events.

Participants who completed the intervention were asked to fill in a Patient Experience Questionnaire during the last measurement session (T3). For this questionnaire, participants had to rate how comfortable they felt with the total number of therapy hours (48 h of additional training), the perceived enjoyment during the ROBUST intervention, the perceived usefulness of ROBUST within their rehabilitation, the perceived added value to their standard rehabilitation, and the perceived improvement in upper limb motor and sensory function on a scale from “Fully disagree” to “Fully agree”. Additionally, participants were asked to select appropriate terms to describe the novel ROBUST intervention and whether they would recommend the program to others using a Visual Analogue Scale ranging from 0 (“Totally not recommending”) to 10 (“Totally recommending”). Furthermore, open-ended questions were included to gather patients’ feedback about the novel intervention.

Additionally, during the first session of each therapy week, the participant filled in the Motivation for Rehabilitation Scale (MORE) [43] to investigate whether the therapy was motivational. This scale ranges from 0 to 119, with a higher score indicating better motivation. This scale is verified as a valid and reliable scale [43].

#### 2.4.3. Multimodal Assessments

To investigate the preliminary effects of this novel integrated intervention, we used a multimodal assessment approach using three types of measurements: clinical, kinematic, and patient-reported outcomes. All assessments were done during each measurement session: baseline (T1), post-control (T2), and post-intervention (T3).

##### Clinical Assessments

The primary outcome of this study was the ARAT [44], evaluating the activity level according to the ICF (International Classification of Functioning, Disability and Health) model [45], measuring grasp, grip, pinch, and gross movement, with a maximum score of 57. Adequate reliability and validity have been reported in the literature [44,46]. The minimal clinically important difference (MCID) is set at 6 points [47].

Other clinical assessments included the TDT, the FMA-UE, and the sARAT. The TDT measures sensory discrimination function with a maximum score of 25 points. Good reliability and validity have been shown [38]. We used 10% of the score range as threshold for the MCID, being 2.5 points. The FMA-UE is a reliable and valid measure of upper limb motor function, with a total score ranging between 0 and 66, whereby higher values indicate better function. The MCID is set at 5.2 points [37]. The sARAT uses the same assessment tasks as the normal ARAT but is done with eyes closed to optimally investigate sensorimotor upper limb function. The total score ranges from 0 to 57. We used 10% of the score range as a threshold for the MCID, being 5.7 points. A good validity is demonstrated [39].

##### Kinematic Assessments

Motor function was assessed using the 4-target Visually Guided Reaching Task (VGR) [48]. Participants performed centre-out reaching movements on the Kinarm robot toward targets as quickly and accurately as possible. Ten parameters (e.g., speed, accuracy, reaction time) were combined into one task score, with lower scores indicating better performance [48,49]. Good reliability and validity have been demonstrated [48,49].

Proprioception was measured using the 9-target Arm Position Matching Task (APM) [50]. The affected arm was passively moved by the Kinarm robot, and participants actively mirrored the position with their less affected arm. Twelve parameters (e.g., magnitude and variability of position errors) were combined into one task score, where lower scores reflect better performance [49,50]. This task also showed good reliability and validity in individuals with stroke [49,50].

Somatosensory discrimination function was investigated with the Passive and Active Discrimination Task. Within the passive condition, the participant’s most affected arm was moved passively by the Kinarm robot in a certain shape, whereafter the participant was asked to remake the figure with the less affected arm, and to discriminate the explored shape from other shapes. Within the active condition, the participant’s most affected arm explored the shape by actively following virtual boundaries built by the robotic device. Again, the less affected arm remade the shape, and the shape was discriminated from others. Both for the passive and active conditions, a factor score was calculated based on the correctness of the answers and the similarity of the remade and explored shapes, whereby a higher score indicated better performance. This assessment has shown good validity and good-to-excellent reliability (not yet published) [51,52].

##### Patient-Reported Outcome

The patient-reported outcome that was used was the Stroke Impairment Scale, especially the Hand subdomain (SIS-Hand) [53]. The SIS-Hand assesses the perceived UL activity by asking participants to indicate the difficulty of five manual activities using their most affected hand (e.g., carrying heavy objects). The total score ranges from 0 to 100, with higher scores indicating better perceived activity. SIS-Hand has good psychometric properties and reported an MCID of 17.8 [54,55].

### 2.5. Data Analysis

Descriptive analysis was used to summarise baseline demographic information of the participants. Regarding potential effectiveness, the major aim was to investigate the number of participants exceeding the available MCID over the intervention period. Additionally, Wilcoxon Signed Rank tests were used to compare the changes during the control period (T1–T2 change) and the intervention period (T2–T3 change). This test was chosen because we compare two related metrics with non-normal distributions. It should be noted that those comparative statistics should be interpreted with caution due to the feasibility nature of the study and the small sample size. For the Wilcoxon Signed Rank Tests comparing the change scores between both periods, effect sizes (r) were calculated as $r=\frac{z}{\sqrt{N}}$ and interpreted following Cohen’s d guidelines (small = 0.10, medium = 0.30, large = 0.50) [56,57,58]. Ninety-five per cent confidence intervals (95%CI) were approximated using Fisher’s z transformation [59]. Specifically, r values were transformed to Fisher’s z, confidence intervals and the standard error (SE = $\frac{1}{\sqrt{N-3}}$) was computed. The resulting limits were back-transformed to the r scale.

To investigate whether motivation changed significantly over the therapy weeks, we conducted a Friedman test.

## 3. Results

**Figure 3:**
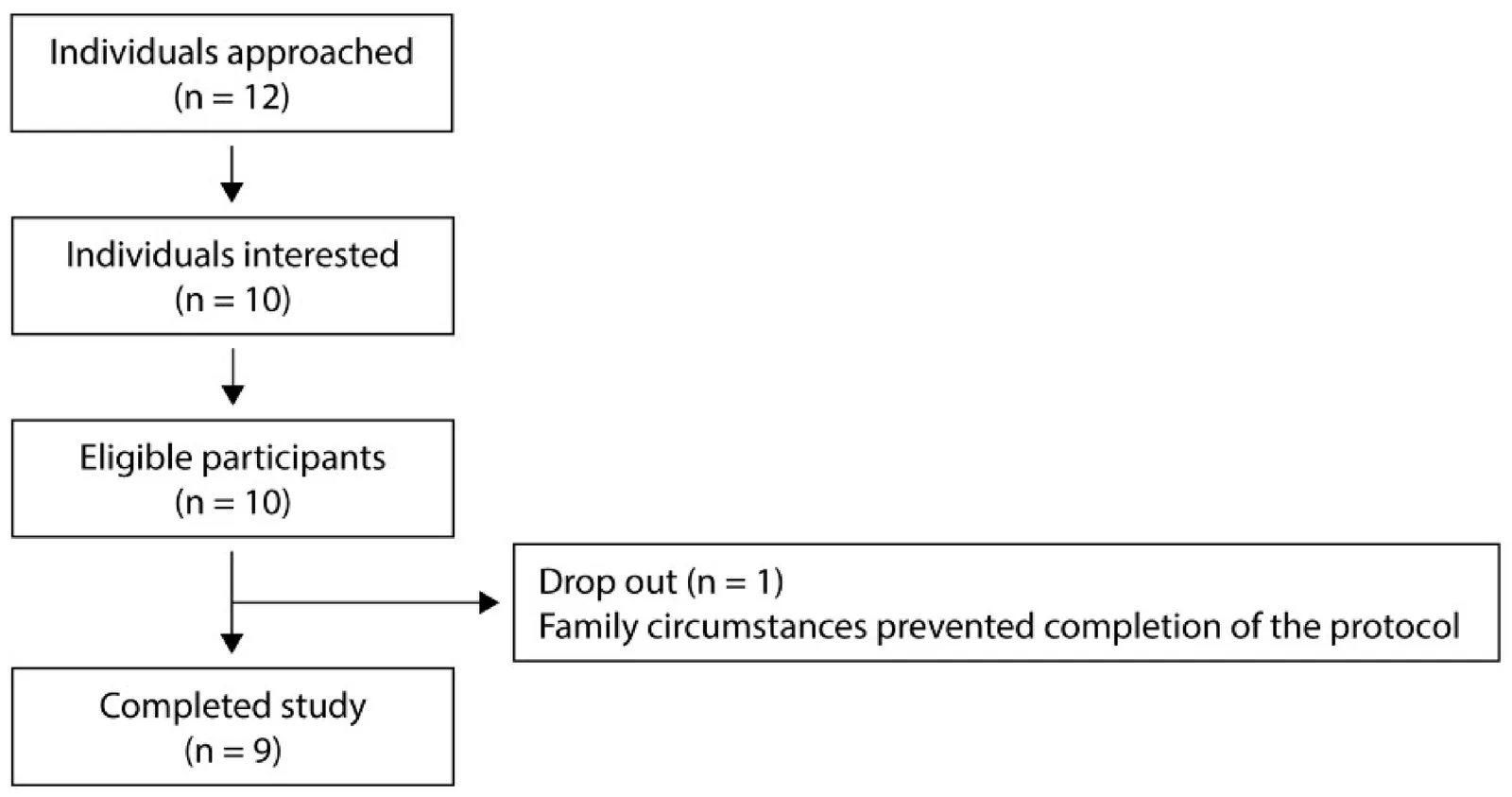
Participant flow diagram.

Between February and August of 2025, 12 individuals were initially contacted. Of these, ten were interested and eligible to participate, resulting in a recruitment rate of 83% (10/12). The participant flow diagram is shown in Figure 3.

### 3.1. Participants’ Characteristics

Ten participants were included in the study, but one participant withdrew at the start because of family circumstances and was therefore not analysed. The baseline characteristics of the nine analysed participants, collected during T1, are presented in Table 2. The participants in this study had a median age of 63 years, with 56% being male. Among the sample, 67% had left hemiparesis and 78% suffered an ischemic stroke. The median time since stroke was 6 years.

### 3.2. Feasibility

Of the ten recruited participants, nine (9/10, 90%) completed the study protocol. One participant discontinued the intervention because of family circumstances.

Of the nine participants who completed the protocol, everyone adhered to the prescribed therapy sessions (9/9, 100%), whereas six adhered to the prescribed home program (6/9, 67%). The three participants who did not reach the goal of training independently for six hours per week reported that the combination of household tasks, scheduled therapy sessions, and other standard care sessions contributed to their lower adherence. On average, participants practised 6.01 h per week, ranging from 3.23 h to 6.70 h.

There were no serious adverse events observed. One participant reported minor neck pain but experienced this as well during household activities before the intervention. One participant reported an increase in ankle clonus sometimes when she was doing the home exercises in a very concentrated way. There were no instances of increased upper limb pain or fatigue reported.

**Figure 4:**
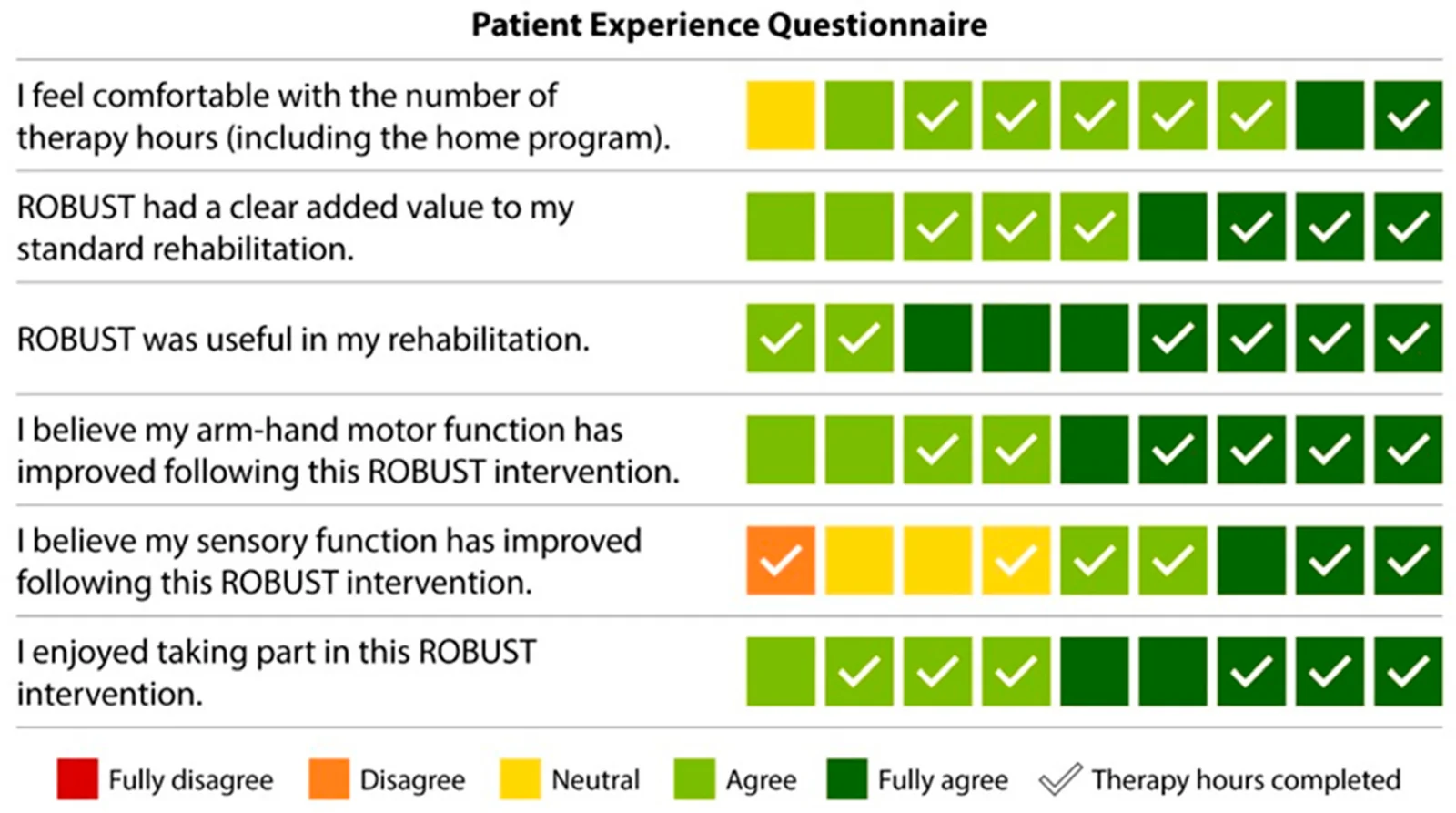
Patient Experience Questionnaire. Each block represents a participant, and participants who adhered to the total prescribed therapy hours are marked with a check.

Based on the Patient Experience Questionnaire, eight participants (8/9, 89%) agreed to fully agreed that they felt comfortable with the total number of therapy hours, whereas one participant (1/9, 11%) was neutral regarding this statement. All participants (9/9, 100%) agreed to fully agreed enjoying the ROBUST intervention, and all (9/9, 100%) agreed to fully agreed that ROBUST has a clear added value and is useful within their standard rehabilitation. All participants (9/9, 100%) had the perception that their arm–hand motor function had improved following the ROBUST intervention, whereas only five (5/9, 56%) agreed to fully agreed that their sensory function improved following ROBUST, three participants (3/9, 33%) were neutral, and one participant (1/9, 11%) disagreed. These results are also shown in Figure 4.

When participants were asked to select terms describing the ROBUST intervention, “motivational”, “feasible”, “effective”, and “unique” were selected. All participants (9/9, 100%) would recommend this ROBUST intervention to others, with a median score of 9 out of 10 and a range between 7.1 and 10. Regarding overall feedback, some participants considered the six-hour-per-week home program too demanding due to practical and organisational constraints. This was particularly the case on days when they also had to attend the scheduled robotic and therapist-led therapy sessions. Positive remarks were the exercise variations, the integrated focus on both sensory and motor functions, the combination of the three therapy types (robotic, therapist-led, home program), and the integration of the functional exercises.

**Figure 5:**
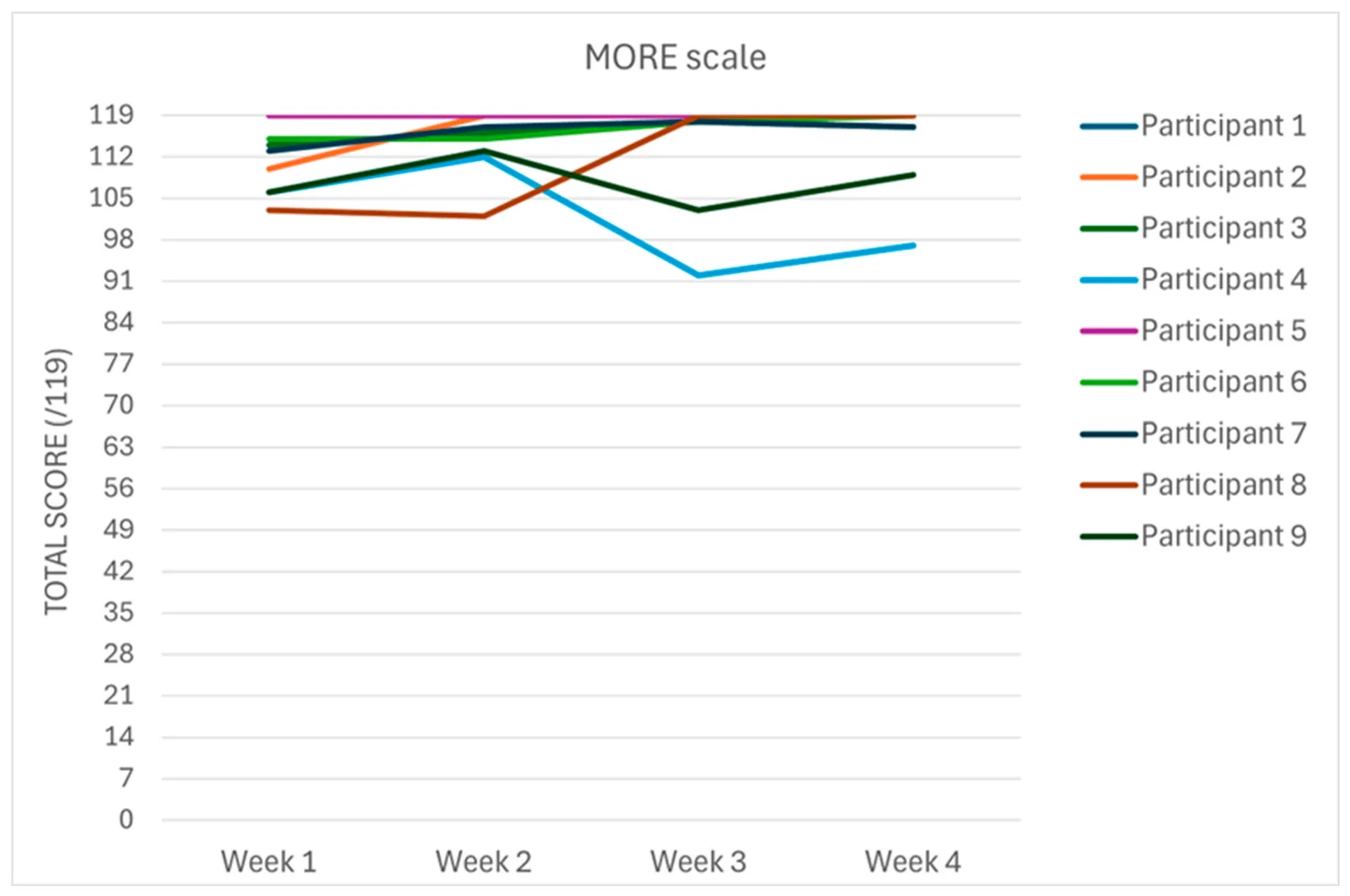
Motivation for Rehabilitation (MORE) scale.

The median score on the Motivation for Rehabilitation (MORE) scale was 116.5 out of 119. A Friedman test indicated no significant difference (*p* = 0.172) between the four weeks of intervention. The result is shown in Figure 5.

### 3.3. Multimodal Assessments

Baseline (T1), post-control (T2), and post-intervention (T3) scores for all assessments are presented in Table 3. The change scores for the control period (T1–T2) and the intervention period (T2–T3), along with their statistical significance and effect sizes, are also reported. There were no significant differences over the control period, except for the Fugl–Meyer Assessment for Upper Extremities (*p* = 0.017).

#### 3.3.1. Clinical Assessments

A median improvement of 7 points was found over the intervention period for the primary outcome (ARAT), exceeding the MCID of 6 points [47]. Furthermore, six participants (6/9, 67%) exceeded this MCID, and there was a significant difference between the change score over the control period and over the intervention period (*p* = 0.012), with a large effect size of r (95%CI) = 0.84 (0.40–0.97).

Furthermore, a median improvement of 9 points on the FMA-UE and 8 points on the sARAT was found, both exceeding the MCID [37,47]. Seven participants (7/9, 78%) reached the MCID for the FMA-UE, whereas eight participants (8/9, 89%) reached it for the sARAT. There was also a significant difference for both scales in change scores between the control period and intervention period, with a large effect size of r (95%CI) = 0.89 (0.55–0.98).

There was no relevant difference found for the TDT. Individual participant change scores for the clinical assessments are shown in Table 3.

#### 3.3.2. Kinematic Assessments

A moderate effect size of r (95%CI) = 0.34 (−0.42–0.82) was found for the comparison of the change scores (control period versus intervention period) for the VGR (motor function), although this was not significant (*p* = 0.051). For the APM (proprioceptive function), a small, non-significant effect was found for the comparison of the change scores (*p* = 0.173, r (95%CI) = 0.22 (−0.52–0.77)). A moderate-to-large effect size was found for the comparison of the change scores of the Passive and Active Discrimination Task, although this was not significant (passive condition: *p* = 0.515, r (95%CI) = 0.65 (−0.02–0.92); active condition: *p* = 0.314, r (95%CI) = 0.45 (−0.30–0.86)).

#### 3.3.3. Patient-Reported Outcome

A median improvement of 25.0 points was found over the intervention period for the SIS-Hand, exceeding the MCID of 17.8 points [55]. Furthermore, six participants (6/9, 67%) exceeded this MCID. A moderate effect size of r (95%CI) = 0.46 (−0.29–0.86) was found for the comparison of the change scores, although this was not significant. Individual participant change scores for the patient-reported outcome are shown in Table 4.

## 4. Discussion

The aim of this study was to investigate the feasibility and preliminary effects of an integrated sensorimotor therapy approach (ROBUST), in addition to standard care, in individuals with chronic stroke. ROBUST combined robotic, therapist-led, and home therapy and focused on sensorimotor functions and overall functionality. Our sample consisted of ten chronic stroke patients with moderate-to-good upper limb motor function, but with remaining upper limb somatosensory function and overall functional impairments.

Feasibility was investigated by recruitment, retention, and adherence rates, as well as the participants’ experiences. The recruitment rate (83%) exceeded the initially expected rate of 31%, which was based on previous studies [20,32,35]. The retention rate was 90%, which was similar to previous studies [20,32,35] and considered good, as guidelines recommend a minimum retention rate of 80% [60]. There was one dropout due to family circumstances.

There was 100% adherence to the on-site therapy sessions and a 67% adherence rate to the home program. This was better than expected, as Essers et al. [35] reported an adherence rate of only 41%, and the other literature shows compliance with home-based therapy ranging from 23% to 64% [61]. One possible explanation for the higher adherence in this current study is that we provided only 24 h of H-GRASP, compared to 48 h over four weeks in Essers et al. [35]. Another explanation might be the combination of the home program with other therapy types, which included face-to-face contact with a therapist three times per week. This may have enhanced motivation and perseverance, as shown by Neibling et al. [42]. One of the six key themes influencing adherence to home-based upper limb practice after stroke is having support and feedback from a therapist. While Essers et al. [35] provided weekly feedback via phone calls, we offered weekly face-to-face feedback, which may have made a difference. Another theme addressed by Neibling et al. [42] was goal setting. To enhance motivation and adherence in this study, we asked the participants to identify personal goals in daily life during the first contact and worked toward those goals during the functional training in each therapy visit.

The three participants who did not adhere to the home program cited the combination of household activities and other scheduled therapy sessions as the main reasons. Therefore, in future studies, the same amount of home therapy (24 h) could be spread over more weeks, reducing the number of hours per week, which may improve adherence.

Most participants (89%) felt comfortable with the total number of therapy hours. Additionally, all participants (100%) enjoyed the ROBUST intervention and agreed that it added clear value to their standard rehabilitation. All participants (100%) perceived improvements in their upper limb motor function, while only 56% perceived improvements in sensory function. This was in line with the outcomes, where we especially found improvements in motor- and activity-related scales, and scales assessing sensorimotor function. The TDT, a sensory assessment, showed the least improvement. A possible explanation for why purely sensory function improved the least is that ROBUST focused primarily on integrated sensorimotor and functional tasks. Pure sensory function was only addressed to a limited extent during the therapist-led sessions. Overall, the participants’ feedback was very positive. They especially mentioned the combination of the three therapy types and the integrated sensorimotor approach as strengths of this novel therapy. In line with the adherence rate, they mentioned that home therapy should be more widespread.

Regarding safety, no serious adverse events occurred. Several factors may explain this favourable safety profile. First, the therapy was delivered by an educated clinician/researcher specialised in neurological rehabilitation. Second, risks were minimised by excluding individuals with uncontrolled epilepsy. Because the Kinarm robot includes a display screen, flashing lights could potentially trigger seizures; carefully defined inclusion and exclusion criteria helped prevent this risk. Third, the therapy was tailored to the personal needs and abilities of each participant, ensuring that exercises were not overly challenging. Finally, the detailed description of the home program and the weekly discussions helped ensure that participants did not practice incorrectly or attempt tasks beyond their capabilities.

To evaluate potential effectiveness, we used clinical, patient-reported, and kinematic outcomes. Our primary aim was to investigate how many participants reached the MCID. For the primary outcome (ARAT), 67% of the participants reached the MCID (i.e., 6 points). Essers et al. [35] found that 11% reached the MCID by following the home program of 48 h. Similarly, 67% reached the MCID (i.e., 17.8 points) on the SIS-Hand (Patient-Reported Outcome), whereas this was 41% in the study of Essers et al. [35]. The combination of the different therapy types and the inclusion of functional training might have enhanced these percentages. Future research may further explore the relative contribution of the different ROBUST components. Also, the FMA-UE and sARAT showed improvements, with 78% and 89% of the participants reaching the MCID, respectively. Because this was a feasibility study, particular emphasis should be placed on individual participant responses, MCID attainment, and effect size estimates rather than on statistical significance. All clinical outcome measures showed change scores over the intervention period with a large effect size (r = 0.84–0.89), except for the TDT (r = 0.02). A possible explanation is that the ROBUST intervention primarily targeted sensorimotor integration and functional activities. In addition, activities requiring specifically tactile (texture) discrimination were not mentioned in the personalised goals of the participants, leading to a higher focus on other components of sensorimotor rehabilitation. Although the TDT has high reliability and validity, it evaluates a very specific type of sensory discrimination function. Only a limited component of the rehabilitation was devoted to purely tactile sensory function, as measured by the TDT, explaining the stability of the score. For the FMA-UE, we found a significant decline during the control phase, which may reflect the measurement error of the FMA-UE between two time points. The significance should be interpreted with caution because of the limited sample size. Page et al. [62] reported that the minimal real difference in the FMA-UE ranges from 4.25 to 7.25 points in persons with chronic stroke and minimal to moderate impairments (such as our sample), and therefore a difference of 3 points might reflect measurement error.

Regarding the kinematic assessments, the Passive and Active Discrimination Task showed a larger effect (r = 0.45–0.65) than the VGR (motor function, r = 0.34) and APM (proprioceptive function, r = 0.22). This is in line with Saenen et al. [32] and may be explained by the fact that the therapy focused more on sensory processing rather than on motor or purely proprioceptive functions.

These potential benefits should be interpreted with caution given the feasibility nature of this study.

### Strengths and Limitations

There are several limitations within this study. First, we used a small sample size, reducing the generalisability and the ability to draw robust conclusions. However, it should be noted that this pilot study with a small sample size was done as an initial step exploring the novel combined intervention, so we could inform feasibility and identify modifications needed in the design of an upcoming pilot clinical trial [63]. There could have been potential observer bias since the therapist providing the therapy sessions was also the assessor. As a result, post-intervention scores may have been rated more favourably (e.g., higher score on ARAT) than would have been the case with an independent blinded assessor. Therefore, a future randomised controlled trial should incorporate blinded outcome assessment to minimise the risk of observer bias. By having four weeks between each measurement session, recall bias was reduced. The detection bias and report bias for the clinical and patient-reported outcomes were minimised by including kinematic measures. Indeed, a strength within this study was the use of a multi-level assessment protocol for a comprehensive evaluation, combining different standardised and recommended measures across all levels of the ICF model. Furthermore, we did not include a separate control group, but by including a 4-week control period before receiving the intervention, participants acted as their own control group. Since the aim of the ROBUST intervention is to serve as an additional therapy, rather than a replacement for the standard care, the control intervention was not dose-matched. Consequently, the current design does not allow for separation of the potential effects of the ROBUST intervention itself from the effects of the substantially increased therapy dose and additional therapist attention received during the intervention period. Several outcomes exceeded the MCID, and relevant effects were observed in clinical, kinematic, and patient-reported outcomes, indicating the potential of our novel integrated therapy program for chronic stroke care.

## 5. Conclusions

In this pilot study, we investigated the feasibility and preliminary effectiveness of an integrated sensorimotor upper limb therapy approach (ROBUST), consisting of robotic, therapist-led, and home therapy. After 48 h, spread over 4 weeks, we found good feasibility, safety, and overall positive participants’ experiences. Furthermore, we found moderate-to-large effects, with most participants exceeding the MCIDs. We suggest future research to evaluate the effectiveness by including a larger sample, blinded assessors, a follow-up period, and a control group to enhance the robustness of the findings.

## Figures and Tables

**Table 1:** Hypothesised relationships between ROBUST components and outcome measures.

ROBUST Component	Intended Mechanism	Outcomes Most Closely Aligned with the Mechanism
Robotic therapy	Proprioceptive processing, Sensorimotor integration	Passive and Active Discrimination Task Arm Position Matching Task Visually Guided Reaching Task
Therapist-led therapy	Sensory processing, Sensorimotor integration, Transfer of sensorimotor gains to meaningful daily activities	Tactile Discrimination Test (sensorimotor) Action Research Arm Test Fugl–Meyer Assessment for Upper Extremity Stroke Impact Scale—Hand subdomain
Home program	Increased practice intensity, Transfer to daily-life arm use	Action Research Arm Test Fugl-Meyer Assessment for Upper Extremity Stroke Impact Scale—Hand subdomain

**Table 2:** Participants’ individual characteristics.

Participant	Age (Years)	Gender	Time After Stroke (Years)	Stroke Type	Most Affected Arm
1	64	Male	3.49	Ischemic	Left
2	50	Male	3.61	Ischemic	Left
3	54	Male	7.38	Haemorrhagic	Left
4	55	Female	1.23	Ischemic	Left
5	75	Female	2.50	Ischemic	Right
6	45	Female	9.88	Ischemic	Right
7	63	Male	8.60	Haemorrhagic	Left
8	68	Male	0.89	Ischemic	Right
9	75	Female	6.37	Ischemic	Left
Median (IQR)	63 (52, 72)	-	6 (3, 9)	-	-
N (%)	-	Male: 5 (56%)	-	Ischemic: 7 (78%)	Left: 6 (67%)

Abbreviations: IQR = interquartile range; N = number of participants.

**Table 3:** Comparison of changes during the control period and the intervention period in clinical, kinematic, and patient-reported measures.

Measure	Control Period (T1–T2 Change)	Intervention Period (T2–T3 Change)	*p*-Value	Effect Size (95%CI)
**Clinical**				
ARAT (/57) *Higher = better*	**0 (−2, 1)**	**7 (4, 9) ^B^**	**0.012**	**0.84 (0.40–0.97)**
TDT (/25) *Higher = better*	1 (−2, 4)	1 (−3, 4)	0.944	0.02 (−0.65–0.68)
FMA-UE (/66) *Higher = better*	**−3 (−4, −1) ^A^**	**9 (6, 13) ^B^**	**0.008**	**0.89 (0.55–0.98)**
sARAT (/57) *Higher = better*	**0 (−3, 1)**	**8 (7, 10) ^B^**	**0.007**	**0.89 (0.55–0.98)**
**Kinematic**				
VGR (Task score) *Lower = better*	−0.17 (−1.35, 0.54)	−0.60 (−1.86, 0.25)	0.051	0.34 (−0.42–0.82)
APM (Task score) *Lower = better*	−0.02 (−0.68, 1.30)	−0.04 (−0.56, 0.09)	0.173	0.22 (−0.52–0.77)
Passive and Active Discrimination Task (Factor score) *Higher = better*				
*Passive condition*	−0.14 (−0.73, 0.22)	0.59 (0.21, 1.08) ^B^	0.515	0.65 (−0.02–0.92)
*Active condition*	0.18 (−0.42, 0.81)	0.56 (0.42, 1.07) ^B^	0.314	0.45 (−0.30–0.86)
**Patient-reported outcome**				
SIS-Hand (/100)	5.0 (−5.0, 17.5)	25.0 (7.5, 30.0) ^B^	0.172	0.46 (−0.29–0.86)

Values are presented as median (first quartile, third quartile). ^A^ Significant difference between T1 and T2 (change over control period); ^B^ Significant difference between T2 and T3 (change over intervention period). Abbreviations: ARAT: Action Research Arm Test; TDT: Tactile Discrimination Test; FMA-UE: Fugl–Meyer Assessment for Upper Extremities; sARAT: sensorimotor Action Research Arm Test; VGR: Visually Guided Reaching Task; APM: Arm Position Matching Task; SIS-Hand: Hand subdomain of Stroke Impact Scale.

**Table 4:** Individual participant change scores for clinical assessments and patient-reported outcomes pre- to post-intervention.

	Clinical Assessments				Patient-Reported Outcome
	ARAT	TDT	FMA-UE	sARAT	SIS-Hand
Participant 1	7 *	5 *	14 *	10 *	30.0 *
Participant 2	11 *	1	7 *	10 *	−10.0
Participant 3	9 *	7 *	10 *	7 *	45.0 *
Participant 4	7 *	−2	12 *	10 *	10.0
Participant 5	8 *	2	13 *	12 *	30.0 *
Participant 6	8 *	2	4	7 *	20.0 *
Participant 7	0	−1	9 *	7 *	25.0 *
Participant 8	5	−6	5	2	30.0 *
Participant 9	3	−3	6 *	9 *	5.0
Median (IQR)	7 * (4, 9)	1 (−3, 4)	9 * (6, 13)	8 * (7, 10)	25.0 * (7.5, 30.0)
Improvements, N (%) *	6 (67%)	2 (22%)	7 (78%)	8 (89%)	6 (67%)

Abbreviations: ARAT: Action Research Arm Test; TDT: Tactile Discrimination Test; FMA-UE: Fugl–Meyer Assessment for Upper Extremities; sARAT: sensorimotor Action Research Arm Test; SIS-Hand: Hand subdomain of Stroke Impact Scale; IQR: interquartile range represented as (first quartile, third quartile); N: number of participants; *: change score higher than the MCID.

## Data Availability

The original data presented in this study are shared under restricted access in the KU Leuven Research Data Repository at [doi:10.48804/U5IZU3]. The data are available upon request for ethical reasons.

## Abbreviations

The following abbreviations are used in this manuscript:

IQR	Interquartile range
ARAT	Action Research Arm Test
TDT	Tactile Discrimination Test
FMA-UE	Fugl–Meyer Assessment for Upper Extremities
sARAT	sensorimotor Action Research Arm Test
VGR	Visually Guided Reaching Task
APM	Arm Position Matching Task
SIS-HAND	Hand subdomain of Stroke Impact Scale

## Appendix A

At the start of the study, participants were asked to provide one to three personalised goals they wanted to improve in their daily lives. Below is an overview of the goals they provided.

**Table A1:** Personalised goals of participants.

Participant	Personalised Goals
Participant 1	- Buttoning a shirt - Unscrewing jar lids
Participant 2	- Using cutlery - Zipping a jacket - Hanging clothes on the coat rack
Participant 3 (drop-out)	- Using cutlery - Stabilising a butter container while spreading - Opening a wine bottle
Participant 4	- Buttoning up the sleeve of a shirt - Unscrewing jar lids - Using cutlery
Participant 5	- Handing over the steering wheel while turning - Activating the automatic turn signal - Adjusting the bicycle gears
Participant 6	- Using cutlery - Steering the wheels of a wheelchair
Participant 7	- Holding a saucepan while stirring - Placing clothes on a high shelf in the wardrobe
Participant 8	- Using cutlery - Holding a yoghurt cup while eating
Participant 9	- Using cutlery - Placing objects on a high shelf in the closet
Participant 10	- Using cutlery - Putting on and taking off a jacket - Brushing hair

## References

[1] Timmis A., Townsend N., Gale C., Grobbee R., Maniadakis N., Flather M., Wilkins E., Wright L., Vos R., Bax J. (2018). European Society of Cardiology: Cardiovascular Disease Statistics 2017. Eur. Heart J..

[2] Meyer S., De Bruyn N., Krumlinde-Sundholm L., Peeters A., Feys H., Thijs V., Verheyden G. (2016). Associations between Sensorimotor Impairments in the Upper Limb at 1 Week and 6 Months after Stroke. J. Neurol. Phys. Ther..

[3] Kitago T., Krakauer J.W. (2013). Motor Learning Principles for Neurorehabilitation. Handbook of Clinical Neurology.

[4] Dromerick A.W., Geed S., Barth J., Brady K., Giannetti M.L., Mitchell A., Edwardson M.A., Tan M.T., Zhou Y., Newport E.L. (2021). Critical Period After Stroke Study (CPASS): A Phase II Clinical Trial Testing an Optimal Time for Motor Recovery after Stroke in Humans. Proc. Natl. Acad. Sci. USA.

[5] Ballester B.R., Maier M., Duff A., Cameirão M., Bermúdez S., Duarte E., Cuxart A., Rodríguez S., San Segundo Mozo R.M., Verschure P.F.M.J. (2019). A Critical Time Window for Recovery Extends beyond One-Year Post-Stroke. J. Neurophysiol..

[6] Wolf S.L., Thompson P.A., Winstein C.J., Miller J.P., Blanton S.R., Nichols-Larsen D.S., Morris D.M., Uswatte G., Taub E., Light K.E. (2010). The EXCITE Stroke Trial: Comparing Early Ad Delayed Constraint-Induced Movement Therapy. Stroke.

[7] Teasell R., Mehta S., Pereira S., McIntyre A., Janzen S., Allen L., Lobo L., Viana R. (2012). Time to Rethink Long-Term Rehabilitation Management of Stroke Patients. Top. Stroke Rehabil..

[8] Ingwersen T., Wolf S., Birke G., Schlemm E., Bartling C., Bender G., Meyer A., Nolte A., Ottes K., Pade O. (2021). Long-Term Recovery of Upper Limb Motor Function and Self-Reported Health: Results from a Multicenter Observational Study 1 Year after Discharge from Rehabilitation. Neurol. Res. Pract..

[9] Borschmann K.N., Hayward K.S. (2020). Recovery of Upper Limb Function Is Greatest Early after Stroke but Does Continue to Improve during the Chronic Phase: A Two-Year, Observational Study. Physiotherapy.

[10] Verheyden G., Nieuwboer A., De Wit L., Thijs V., Dobbelaere J., Devos H., Severijns D., Vanbeveren S., De Weerdt W. (2008). Time Course of Trunk, Arm, Leg, and Functional Recovery after Ischemic Stroke. Neurorehabilit. Neural Repair.

[11] Zandvliet S.B., Kwakkel G., Nijland R.H.M., van Wegen E.E.H., Meskers C.G.M. (2020). Is Recovery of Somatosensory Impairment Conditional for Upper-Limb Motor Recovery Early After Stroke?. Neurorehabil. Neural Repair.

[12] Purton J., Sim J., Hunter S.M. (2023). Stroke Survivors’ Views on Their Priorities for Upper-Limb Recovery and the Availability of Therapy Services after Stroke: A Longitudinal, Phenomenological Study. Disabil. Rehabil..

[13] Meyer S., Verheyden G., Brinkmann N., Dejaeger E., De Weerdt W., Feys H., Gantenbein A.R., Jenni W., Laenen A., Lincoln N. (2015). Functional and Motor Outcome 5 Years after Stroke Is Equivalent to Outcome at 2 Months: Follow-Up of the Collaborative Evaluation of Rehabilitation in Stroke Across Europe. Stroke.

[14] Carlsson H., Gard G., Brogårdh C. (2018). Upper-Limb Sensory Impairments after Stroke: Self-Reported Experiences of Daily Life and Rehabilitation. J. Rehabil. Med..

[15] Carlsson H. (2022). Sensory Impairment of the Upper Limb after Stroke: Influence on Daily Life and Effects of a Novel Training Approach. Ph.D. Thesis.

[16] Sidarta A., Lim Y.C., Wong R.A., Tan I.O., Kuah C.W.K., Ang W.T. (2022). Current Clinical Practice in Managing Somatosensory Impairments and the Use of Technology in Stroke Rehabilitation. PLoS ONE.

[17] Carlsson H., Rosén B., Björkman A., Pessah-Rasmussen H., Brogårdh C. (2021). SENSory Re-Learning of the UPPer Limb (SENSUPP) after Stroke: Development and Description of a Novel Intervention Using the TIDieR Checklist. Trials.

[18] Torre K., Hammami N., Metrot J., Van Dokkum L., Coroian F., Mottet D., Amri M., Laffont I. (2013). Somatosensory-Related Limitations for Bimanual Coordination after Stroke. Neurorehabil. Neural Repair.

[19] Lv Q., Zhang J., Pan Y., Liu X., Miao L., Peng J., Song L., Zou Y., Chen X. (2022). Somatosensory Deficits After Stroke: Insights From MRI Studies. Front. Neurol..

[20] De Bruyn N., Saenen L., Thijs L., Van Gils A., Ceulemans E., Essers B., Lafosse C., Michielsen M., Beyens H., Schillebeeckx F. (2020). Sensorimotor vs. Motor Upper Limb Therapy for Patients with Motor and Somatosensory Deficits: A Randomized Controlled Trial in the Early Rehabilitation Phase After Stroke. Front. Neurol..

[21] Rand D. (2018). Proprioception Deficits in Chronic Stroke—Upper Extremity Function and Daily Living. PLoS ONE.

[22] Carey L.M., Matyas T.A., Baum C. (2018). Effects of Somatosensory Impairment on Participation after Stroke. Am. J. Occup. Ther..

[23] Asan A.S., McIntosh J.R., Carmel J.B. (2022). Targeting Sensory and Motor Integration for Recovery of Movement After CNS Injury. Front. Neurosci..

[24] Pumpa L.U., Cahill L.S., Carey L.M. (2015). Somatosensory Assessment and Treatment after Stroke: An Evidence-Practice Gap. Aust. Occup. Ther. J..

[25] Van Gils A., Zou Y., Meyer S., Michielsen M., Lafosse C., Beyens H., Schillebeeckx F., Kos D., Verheyden G. (2025). Tracking Bimanual Recovery after Stroke: Grasp Function and Stroke Severity Predict 1-Year Performance. Clin. Rehabil..

[26] Chen X., Liu F., Yan Z., Cheng S., Liu X., Li H., Li Z. (2018). Therapeutic Effects of Sensory Input Training on Motor Function Rehabilitation after Stroke. Medicine.

[27] Carey L., Macdonell R., Matyas T.A. (2011). SENSe: Study of the Effectiveness of Neurorehabilitation on Sensation: A Randomized Controlled Trial. Neurorehabil. Neural Repair.

[28] Carey L. Evidence-Based SENSe Therapy. https://sensetherapy.net.au/health-professionals/evidence-based-sense-therapy/.

[29] Winter L., Huang Q., Sertic J.V.L., Konczak J. (2022). The Effectiveness of Proprioceptive Training for Improving Motor Performance and Motor Dysfunction: A Systematic Review. Front. Rehabil. Sci..

[30] Carey L. SENSe Therapy Principles. https://sensetherapy.net.au/sense-therapy/sense-therapy-principles/.

[31] Wang H., Wu X., Li Y., Yu S. (2025). Efficacy of Robot-Assisted Training on Upper Limb Motor Function After Stroke: A Systematic Review and Network Meta-Analysis. Arch. Rehabil. Res. Clin. Transl..

[32] Saenen L., Orban de Xivry J.-J., Verheyden G. (2022). Upper Limb Sensory Processing: Development of a Robotic Paradigm. Ph.D. Thesis.

[33] Toh S.F.M., Chia P.F., Fong K.N.K. (2022). Effectiveness of Home-Based Upper Limb Rehabilitation in Stroke Survivors: A Systematic Review and Meta-Analysis. Front. Neurol..

[34] Simpson L.A., Eng J.J., Chan M. (2017). H-GRASP: The Feasibility of an Upper Limb Home Exercise Program Monitored by Phone for Individuals Post Stroke. Disabil. Rehabil..

[35] Essers B., Veerbeek J.M., Luft A.R., Verheyden G. (2024). The Feasibility of the Adapted H-GRASP Program for Perceived and Actual Daily-Life Upper Limb Activity in the Chronic Phase Post-Stroke. Disabil. Rehabil..

[36] Salbach N.M., Yao J.K., Lindsay M.P., Nelson M.L.A., Shi J., O’Connell C., Barclay R., Bastasi D., Boulos M.I., Boyce J. (2025). Canadian Stroke Best Practice Recommendations Rehabilitation, Recovery, and Community Participation Following Stroke, Part Two: Delivery of Stroke Rehabilitation to Optimize Functional Recovery, 7th Edition Update 2025. Am. J. Phys. Med. Rehabil..

[37] Lin J.-H., Hsu M.-J., Sheu C.-F., Wu T.-S., Lin R.-T., Chen C.-H., Hsieh C.-L. (2009). Psychometric Comparisons of 4 Measures for Assessing Upper-Extremity Function in People With Stroke. Phys. Ther..

[38] Carey L.M., Oke L.E., Matyas T.A. (1997). Impaired Touch Discrimination After Stroke: A Quantiative Test. Neurorehabil. Neural Repair.

[39] Saenen L., De Bruyn N., Verheyden G. (2024). Validity of a Sensorimotor Adaptation of the Action Research Arm Test (SARAT) in Chronic Stroke. Disabil. Rehabil..

[40] Borg G. (1998). Borg’s Perceived Exertion and Pain Scales.

[41] Keeling A.B., Piitz M., Semrau J.A., Hill M.D., Scott S.H., Dukelow S.P. (2021). Robot Enhanced Stroke Therapy Optimizes Rehabilitation (RESTORE): A Pilot Study. J. Neuroeng. Rehabil..

[42] Neibling B., Hayward K.S., Smith M., Chapman P., Barker R.N. (2024). Perseverance with Home-Based Upper Limb Practice after Stroke: Perspectives of Stroke Survivors and Their Significant Others. Disabil. Rehabil..

[43] Yoshida T., Otaka Y., Kitamura S., Ushizawa K., Kumagai M., Kurihara Y., Yaeda J., Osu R. (2022). Development and Validation of New Evaluation Scale for Measuring Stroke Patients’ Motivation for Rehabilitation in Rehabilitation Wards. PLoS ONE.

[44] Yozbatiran N., Der-Yeghiaian L., Cramer S.C. (2008). A Standardized Approach to Performing the Action Research Arm Test. Neurorehabil. Neural Repair.

[45] World Health Organization (2002). Towards a Common Language for Functioning, Disability, and Health: ICF.

[46] van der Lee J.H., de Groot V., Beckerman H., Wagenaar R.C., Lankhorst G.J., Bouter L.M. (2001). The Intra- and Interrater Reliability of the Action Research Arm Test: A Practical Test of Upper Extremity Function in Patients with Stroke. Arch. Phys. Med. Rehabil..

[47] Connell L.A., Tyson S.F. (2012). Clinical Reality of Measuring Upper-Limb Ability in Neurologic Conditions: A Systematic Review. Arch. Phys. Med. Rehabil..

[48] Coderre A.M., Zeid A.A., Dukelow S.P., Demmer M.J., Moore K.D., Demers M.J., Bretzke H., Herter T.M., Glasgow J.I., Norman K.E. (2010). Assessment of Upper-Limb Sensorimotor Function of Subacute Stroke Patients Using Visually Guided Reaching. Neurorehabil. Neural Repair.

[49] BKIN Technologies Ltd. Kinarm Standard Tests. https://kinarm.com/solutions/kinarm-standard-tests/.

[50] Dukelow S.P., Herter T.M., Moore K.D., Demers M.J., Glasgow J.I., Bagg S.D., Norman K.E., Scott S.H. (2010). Quantitative Assessment of Limb Position Sense Following Stroke. Neurorehabil. Neural Repair.

[51] Saenen L., Orban de Xivry J.J., Verheyden G. (2022). Development and Validation of a Novel Robot-Based Assessment of Upper Limb Sensory Processing in Chronic Stroke. Brain Sci..

[52] Heremans C., Saenen L., Shetty C., Orban de Xivry J.-J., Verheyden G. (2025). Reliability and Validity of a New Robot-Based Upper Limb Sensory Processing Assessment After Stroke.

[53] Duncan P.W., Bode R.K., Min Lai S., Perera S. (2003). Rasch Analysis of a New Stroke-Specific Outcome Scale: The Stroke Impact Scale11No Commercial Party Having a Direct Financial Interest in the Results of the Research Supporting This Article Has or Will Confer a Benefit upon the Author(s) or upon Any Organization with Which the Author(s) Is/Are Associated. Arch. Phys. Med. Rehabil..

[54] Vellone E., Savini S., Fida R., Dickson V.V., Melkus G.D., Carod-Artal F.J., Rocco G., Alvaro R. (2015). Psychometric Evaluation of the Stroke Impact Scale 3.0. J. Cardiovasc. Nurs..

[55] Lin K.-C., Fu T., Wu C.-Y., Hsieh Y.-W., Chen C.-L., Lee P.-C. (2010). Psychometric Comparisons of the Stroke Impact Scale 3.0 and Stroke-Specific Quality of Life Scale. Qual. Life Res..

[56] Ivarsson A., Andersen M.B., Johnson U., Lindwall M. (2013). To Adjust or Not Adjust: Nonparametric Effect Sizes, Confidence Intervals, and Real-World Meaning. Psychol. Sport Exerc..

[57] Cohen J. (1992). A Power Primer. Psychol. Bull..

[58] Sullivan G.M., Feinn R. (2012). Using Effect Size—Or Why the P Value Is Not Enough. J. Grad. Med. Educ..

[59] (2007). Fisher’s Z Transformation. Encyclopedia of Measurement and Statistics.

[60] Teresi J.A., Yu X., Stewart A.L., Hays R.D. (2022). Guidelines for Designing and Evaluating Feasibility Pilot Studies. Med. Care.

[61] Miller K.K., Porter R.E., DeBaun-Sprague E., Van Puymbroeck M., Schmid A.A. (2017). Exercise after Stroke: Patient Adherence and Beliefs after Discharge from Rehabilitation. Top. Stroke Rehabil..

[62] Page S.J., Fulk G.D., Boyne P. (2012). Clinically Important Differences for the Upper-Extremity Fugl-Meyer Scale in People With Minimal to Moderate Impairment Due to Chronic Stroke. Phys. Ther..

[63] Leon A.C., Davis L.L., Kraemer H.C. (2011). The Role and Interpretation of Pilot Studies in Clinical Research. J. Psychiatr. Res..

